# The 125th Lys and 145th Thr Amino Acids in the GTPase Domain of Goose Mx Confer Its Antiviral Activity against the Tembusu Virus

**DOI:** 10.3390/v10070361

**Published:** 2018-07-06

**Authors:** Shun Chen, Miao Zeng, Peng Liu, Chao Yang, Mingshu Wang, Renyong Jia, Dekang Zhu, Mafeng Liu, Qiao Yang, Ying Wu, Xinxin Zhao, Anchun Cheng

**Affiliations:** 1 Institute of Preventive Veterinary Medicine, Sichuan Agricultural University, No. 211 Huimin Road, Wenjiang District, Chengdu 611130, Sichuan, China; ZM_Alicen@163.com (M.Z.); lxpliupeng@126.com (P.L.); 15108224618@163.com (C.Y.); mshwang@163.com (M.W.); cqrc_jry@163.com (R.J.); liumafengra@163.com (M.L.); yangqiao721521@sina.com (Q.Y.); yingzi_no1@126.com (Y.W.); xxinzhao@sicau.edu.cn (X.Z.); 2 Research Center of Avian Disease, College of Veterinary Medicine, Sichuan Agricultural University, Chengdu 611130, Sichuan, China; zdk24@sicau.edu.cn; 3 Key Laboratory of Animal Disease and Human Health of Sichuan Province, Sichuan Agricultural University, Chengdu 611130, Sichuan, China

**Keywords:** TMUV, Mx, antiviral activity, GTP-binding elements, L4 loop

## Abstract

The Tembusu virus (TMUV) is an avian pathogenic flavivirus that causes a highly contagious disease and catastrophic losses to the poultry industry. The myxovirus resistance protein (Mx) of innate immune effectors is a key antiviral “workhorse” of the interferon (IFN) system. Although mammalian Mx resistance against myxovirus and retrovirus was witnessed for decades, whether or not bird Mx has anti-flavivirus activity remains unknown. In this study, we found that the transcription of goose Mx (goMx) was obviously driven by TMUV infection, both in vivo and in vitro, and that the titers and copies of TMUV were significantly reduced by goMx overexpression. In both primary (goose embryo fibroblasts, GEFs) and passaged cells (baby hamster kidney cells, BHK21, and human fetal kidney cells, HEK 293T), it was shown that goMx was mainly located in the cytoplasm, and sporadically distributed in the nucleus. The intracellular localization of this protein is attributed to the predicted bipartite nuclear localization signal (NLS; 30 residues: the 441st–471st amino acids of goMx). Intuitively, it seems that the cells with a higher level of goMx expression tend to have lower TMUV loads in the cytoplasm, as determined by an immunofluorescence assay. To further explore the antiviral determinants, a panel of variants was constructed. Two amino acids at the 125th (Lys) and 145th (Thr) positions in GTP-binding elements, not in the L4 loop (40 residues: the 532nd–572nd amino acids of goMx), were vital for the antiviral function of goMx against TMUV in vitro. These findings will contribute to our understanding of the functional significance of the antiviral system in aquatic birds, and the development of goMx could be a valuable therapeutic agent against TMUV.

## 1. Introduction

The myxovirus resistance protein (Mx) is an interferon-induced GTPase that blocks the replication of both DNA and RNA viruses [1,2]. The Mx gene is highly polymorphic in most species, resulting in different antiviral properties [3,4]. Previous studies indicated that a specific single nucleotide polymorphism (SNP) in the pig Mx2 or chicken Mx gene is responsible for its antiviral function, i.e., an SNP at the 514th amino acid (aa) of porcine Mx2 and at the 631st aa of chicken Mx determine the antiviral activity [4,5]. It is generally believed that two Mx homologs (Mx1 and Mx2, also called MxA and MxB in human, respectively) exist in humans, rodents, and mammals. The paramount role of rodent Mx1 and human MxA or MxB is well illustrated [1,6,7]. Unlike the mammalian Mx system, birds have only one Mx homolog. However, the antiviral properties and determinants of Mx against flaviviruses are unknown.

The Mx protein is assumed to consist of an N-terminal G domain, a bundle signaling element (BSE), and a C-terminal antiparallel four-helical bundle, designated the stalk domain [1]. The conserved tripartite GTP-binding elements in the G domain can bind and hydrolyze GTP, and then trigger large-scale conformational movement via the adjacent BSE [8,9]. The stalk domain mediates the assembly of ring-like Mx oligomers [10,11], while residues in the L4 loop of the helical stalk domain, shown as signatures of strong positive selection, were proven to serve as major viral target-specificity determinants, suggesting their potential roles in the antiviral interface during the host–virus interaction [10,12]. The oligomeric form of Mx, mediated via the BSE–stalk interface, is invariably in a storage form [8,13], whereas the dimerization of Mx via the GTPase domain interfaces is transient [9,14,15], indicating that the intra- and intermolecular domain interactions involved in core motifs of Mx are required for viral target recognition and antiviral activity.

The cytoplasmic Mx protein associates with intracellular membranes known as coat protein I (COPI)-positive membranes of the smooth endoplasmic reticulum/Golgi-intermediate compartment [16], and it assembles into a ring-like structure around negatively charged liposomes using a lysine-rich stretch of the L4 loop, known as the lipid-binding moiety [17]. The cytoplasmic human MxA protein can inhibit the West Nile virus (WNV) by sequestering the capsid protein and reducing titers of secreted WNV particles [18]. The nuclear rodent Mx1 proteins inhibit viruses that have a nuclear replication phase, such as influenza and other influenza-like viruses [1,19]. Furthermore, human MxA, artificially designed to locate in the nucleus, inhibits the transcription step of the influenza virus genome [20,21]. Generally, the Mx protein redistributes to sites of viral replication, and promotes missorting of essential viral constituents upon virus infection. However, whether the antiviral properties of the single bird Mx are more closely related to the homologous Mx1 or Mx2 remains unknown.

The Tembusu virus (TMUV), an emerging pathogen within the group *Ntaya,* genus *Flavivirus*, can plague domestic ducks and geese, and was potentially overlooked regarding its zoonotic transmission potential. Characterized by an ovarian hemorrhage and neurological signs in almost all diseased birds, the TMUV disease can cause infection and morbidity rates that are typically high (up to 90 %), and mortality rates as high as 30% [22,23]. The TMUV infection can also significantly upregulate the expression of type I and type III interferons (IFNs) and some critical IFN-stimulated genes (ISGs), both in vivo and in vitro [24,25]. Currently, a few vaccine candidates are proposed [26,27,28], and the strategy also focuses on the development of replication inhibitors designed to affect the enzyme activity of viral proteins, such as non-structural proteins 3 and 5 (NS3 and NS5) [29]. Among all the ISGs, the interferon-inducible transmembrane protein (IFITM) and the 2′-5′-oligoadenylate synthetase (OAS) families were well demonstrated as antivirals against the TMUV in birds [30,31].

Mx genes of ducks and chickens were cloned and identified previously. In ducks, Mx proteins failed to inhibit influenza virus replication [32]. Furthermore, the chicken Mx protein showed conflicting evidence for its antiviral properties owing to its polymorphisms [33]. Based on our previous work [34,35], the immune characteristics, subcellular location, and key amino acids for the antiviral ability of goose Mx (goMx) against TMUV were studied. Collectively, these findings will contribute to our understanding of the functional features of goMx, and to the development of novel therapeutic approaches against viral disease.

## 2. Materials and Methods

### 2.1. Cells, Viruses, and Animals

Primary goose embryo fibroblasts (GEFs) were derived from ten-day-old goose embryos, and were maintained in Dulbecco’s modified Eagle’s medium (DMEM; TransGen Biotech, Beijing, China) supplemented with 10% newborn calf serum (NCBS; HyClone, Logan, UT, USA) and grown at 37 °C. Baby hamster kidney cells (BHK21) and human fetal kidney cells (HEK 293T) were cultivated in DMEM with 5% fetal bovine serum (FBS; HyClone), at 37 °C with 5% CO_2_. The TMUV CQ strain (accession: KM233707) was used and its 50% tissue culture infection dose (TCID_50_) was detected in GEFs as 6.3 × 10^6^ TCID_50_/0.1 mL [36]. All one-week-old healthy goslings were purchased from the breeding center of Sichuan Agricultural University, Yaan City, China, and then maintained and observed for three days in laboratory animal rooms prior to experiments. The welfare of the animals was ensured during the sampling process.

### 2.2. Immunological Characteristic Research In Vitro

The polyinosinic polycytidylic acid (Poly(I:C); Sigma, St. Louis, MO, USA) can mimic the treatment of double-stranded RNA. Poly(I:C) (30 mg/mL; 50 μL/well) and TMUV (1000 TCID_50_; 100 μL/well) were used to treat the GEFs seeded in a 12-well plate. The negative controls were treated with the same volume of PBS. Three repeats of each group were collected at the indicated time points (3 h, 12 h, 24 h, and 36 h) for qRT-PCR detection of the messenger RNA (mRNA) expression level of the goMx gene.

### 2.3. The Effects of Viruses on goMx mRNA Levels In Vivo

For the viral challenge experiment, goslings were randomly divided into four groups. Four individuals from each group were injected intramuscularly with TMUV (1 μL of TMUV per gram of body weight). The birds in the mock groups were injected with PBS. Afterward, six tissues, including brains (B), blood (BL), liver (LI), pancreas (P), spleen (SP), and thymus (T), were obtained from each individual for the detection of goMx mRNA expression levels.

### 2.4. Plasmid Construction

To eliminate the influence of the enhanced GFP (EGFP) protein on the structure and function of goMx, we introduced the hydrophobicity linker peptide (GGGGS)_3_ into pEGFP-C1 at the BglII and KpnI sites to construct fusion proteins with an N-terminal EGFP moiety (pEGFP-C1-Mx). The nuclear localization signals (NLSs) of the goMx sequence were predicted using cNLS Mapper (http://nls-mapper.iab.keio.ac.jp/cgi-bin/NLS_Mapper_form.cgi) by calculating NLS scores. Based on the prediction, the bipartite NLS sequence of goMx was fused to the pEGFP-C1 to construct the pEGFP-C1-NLS/Mx plasmid, and the classical NLS of the SV40 large T antigen was constructed as pEGFP-C1-SV40, serving as a positive control.

The construction and expression of wild-type goMx and its variants were described previously [35]. Those expression plasmids were expressed as C-terminal six-His-tag fusions from pcDNA3.1(+) expression plasmids which facilitate detection via anti-His monoclonal antibody (mAb; Abcam, Cambridge, MA, USA). Site-directed mutagenesis and deletion variant constructions were carried out using the overlap-PCR method. To create the GTPase-deficient mutants, *Mx/K125A and *Mx/T145A, the codon AAA encoding Lys (K) at the 125th (K125) and the codon ACT encoding Thr (T) at 145th (T145) of goMx were both converted to the GCC codon of Ala using the indicated primers (Table 1). The *Mx /ΔL4 deletion plasmid was constructed similarly using the indicated primers (Table 1). The two combination mutation plasmids of K125A*Mx/ΔL4 and T145A*Mx/ΔL4 were constructed based on the *Mx/K125A and *Mx/T145A substitution mutations, respectively.

### 2.5. Intracellular Location

Monolayer GEFs, BHK21, and HEK 293T cells were seeded on coverslips overnight, and were then transfected with a test group of pEGFP-C1-Mx and pEGFP-C1-NLS/Mx (pEGFP-C1 and pEGFP-C1-SV40 as controls, respectively) by using the EL transfection reagent (TransGen, Beijing, China) according to the manufacturer’s directions. After 24 h of transfection, the micro coverslips were directly fixed in 4% paraformaldehyde, and were stained with 4′,6-diamidine-2′-phenylindole dihydrochloride (DAPI) to determine the location of pEGFP-C1-Mx or pEGFP-C1-NLS/Mx. For pcDNA3.1(+) carrying His-tagged goMx or its variants, BHK21 cells were transfected with indicated expression plasmids. At 24 h post-transfection, cells were fixed, permeated, and stained with a mouse His-tagged mAb (as mentioned above) at a 1:500 dilution, and were then incubated with the Alexa Fluor PE-conjugated fluorescent goat-anti-mouse secondary antibodies (Life Technologies, Carlsbad, CA, USA).

### 2.6. Subcellular Colocalization of goMx with the TMUV In Vitro

The purified and concentrated TMUV was prepared as antigen and immunized mice for production of a mouse polyclonal antibody. To detect the goMx pattern upon TMUV infection, GEFs were transfected with pEGFP-C1-Mx expression plasmids for 18 h, and were then infected with 100 μL of 1000 TCID_50_ TMUV for another 24 h. Subsequently, the cells were washed in PBS, fixed in 4% paraformaldehyde for 20 min at 4 °C, permeabilized with 0.2% Triton for 30 min, and then blocked with 5% bovine serum albumin (BSA; Beyotime, Shanghai, China) overnight. The coverslips were incubated with the home-made mouse polyclonal antibody (at 1:500 dilution) which could target the whole TMUV protein in 1% BSA PBS for 1 h, washed in PBS for three, and then incubated with Alexa Fluor PE-conjugated fluorescent goat-anti-mouse secondary antibodies for an additional 45 min. The coverslips were then washed again in PBS and mounted on slides. The fluorescence of the cells was acquired by microscopy (Nikon Eclipse 80i; Tokyo, Japan).

### 2.7. Antiviral Activity Assay of goMx

GEFs in 12-well plates were transiently transfected with wild-type goMx or its variants at 1.6 μg per well, followed by infection with 100 μL of 1000 TCID_50_ TMUV at 24 h post-transfection (hpt; 18 hpt for pEGFP-C1-Mx and pEGFP-C1groups), and then, the cell lysates were resuspended using 500 μL of PBS at 24 h, 48 h, and 72 h after infection (hpi). The collected suspension was used for determining TMUV TCID_50_ and copies. Total RNA was purified using a TIANamp Virus DNA/RNA Kit (Tiangen, Beijing, China), and was instantly used for reverse transcription. The viral copies of TMUV-infected GEFs were quantified with RT-qPCR using the standard curve, Y = 39.995 − 3.529logX. For each time point, three independent assays were performed, and each sample was analyzed in triplicate. Additionally, the TCID_50_ assay was performed in a 96-well plate with monolayer GEF cells infected with 0.1 mL of ten-fold serial dilutions of the viral samples. After 96 h of incubation at 37 °C, the titers of the virus were calculated using the standard method of Reed and Muench.

### 2.8. Real-Time Fluorescence Quantitative PCR (RT-qPCR)

RT-qPCR was conducted in a CFX96 TM Real-Time PCR Detection System (Bio-Rad, USA) to quantify goMx transcript levels and TMUV copies. Total RNA was extracted using RNAiso Plus according to the manufacturer’s instructions (TaKaRa, Dalian, China). RNA concentrations were determined using a Thermo Scientific NanoDrop 2000. An equal quantity of 1 μg complementary DNA (cDNA) was instantly synthesized using a HiScript^1st^ Strand cDNA Synthesis Kit (Vazyme, Nanjing, USA), and was conserved at −80 °C until use. Transcript levels of goMx were measured with RT-qPCR using the specific primers as previously reported [35]. The data were analyzed by the 2^−ΔΔCt^ method, and expression values were normalized to glyceraldehyde 3-phosphate dehydrogenase (GAPDH) mRNA levels in the same samples.

### 2.9. Western Blot Analysis

An equal volume of samples was boiled for 10 min, and proteins were separated by 12% SDS–PAGE, and transferred to a polyvinylidene fluoride (PVDF) membrane (Millipore, Bedford, MA, USA) via a wet transfer method. The membranes were blocked with TBST buffer (10 mM Tris-HCl (pH 8.0), 0.15 M NaCl, and 0.05% Tween-20) containing 5% skim milk overnight, and were then incubated with anti-His (Abcam, USA) or EGFP-tagged monoclonal antibody (mAb) as the primary antibody and mouse anti-β-actin (Ruiying Biological, Suzhou, China) at a 1:2000 dilution for 2 h, followed by incubation with goat anti-mouse immunoglobulin G (IgG) horseradish peroxidase (HRP)-conjugated antibody for 1 h (Earthox, San Francisco, CA, USA) at a 1:2000 dilution for 1 h. Afterward, detection was performed using enhanced chemiluminescence (ECL; Bio-Rad, Hercules, CA, USA).

### 2.10. Cell Viability Analysis

GEFs were seeded in 96-well microplates in three replicates, and were transfected with goMx and its variants. At 48 h post-transfection, a CCK8 solution (Beyotime, Shanghai, China) was added to the wells, which were incubated for an additional 3 h at 37 °C. Optical density was measured using a Thermo Scientific Varioskan Flash (Thermo, Hercules, CA, USA) at a wavelength of 450 nm.

### 2.11. Statistics

All data were analyzed with GraphPad Prism 7 (GraphPad Software Inc., San Diego, CA, USA), and are presented as the means ± standard deviation (SD). A Student’s *t*-test was used to determine the significance of differences between treated and control groups. A *p*-value <0.05 was considered statistically significant.

## 3. Results

### 3.1. TMUV Can Stimulate the goMx Gene both In Vitro and In Vivo

**Figure 1:**
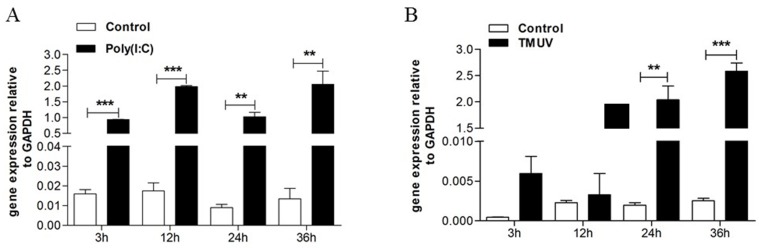
Time course study of goose myxovirus resistance protein (goMx) messenger RNA (mRNA) expression upon polyinosinic polycytidylic acid (Poly(I:C)) stimulation and Tembusu virus (TMUV) infection. Goose embryo fibroblast (GEF) cells were either induced with Poly(I:C) (30 mg/mL; 50 μL/well) or TMUV (1000 50% tissue culture infection dose (TCID_50_); 100 μL/well), and total RNA was harvested at designated time points and reverse transcribed into complementary DNA (cDNA). The resultant cDNA was used as the template in a quantitative PCR reaction, using primers specific for goMx, and glyceraldehyde 3-phosphate dehydrogenase (GAPDH) was used as an internal reference. (**A**) The mRNA expression levels of goMx against Poly(I:C) stimulation in GEF. (**B**) Analyses of goMx transcript levels post-TMUV infection in GEF. Each time point was assayed in triplicate, with error bars given as mean ± SD. Error bars indicate standard error. Asterisks (*) mark the significant difference between experimental and control groups (** *p* < 0.01; *** *p* < 0.001).

**Figure 2:**
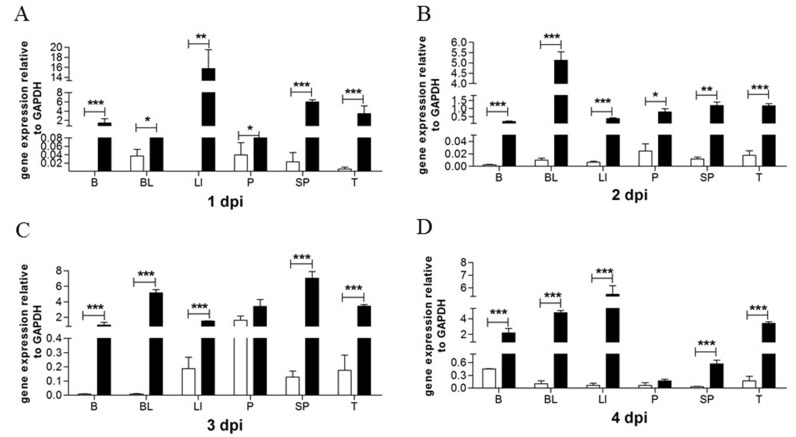
Tissue-specific mRNA of goMx in TMUV-infected goslings. The white columns are for control groups, while the black columns stand for test groups. Goslings were infected with TMUV (1 μL volume of TMUV per gram of the body weight), then tissues were collected, including brains (B), blood (BL), liver (LI), pancreas (P), spleen (SP), and thymus (T). GoMx transcript detection at one day (**A**), two days post infection (**B**), three days (**C**) and four days (**D**) post infection. GAPDH was employed as an internal reference. Asterisks (*) mark the significant difference between experimental and control groups (* *p* < 0.05; ** *p* < 0.01; *** *p* < 0.001). Error bars indicate standard error.

GEFs were stimulated with Poly(I:C) and TMUV. As expected, the upregulated transcripts of the goMx gene were detected after Poly(I:C) treatment at every time point (Figure 1A). In general, the goMx mRNA levels also increased as early as 3 h, and were significantly induced at 24 h post-TMUV infection (Figure 1B). To further identify the role of the goMx in TMUV infection in vivo, total RNA was extracted separately from TMUV-infected goslings or mock goslings. The goMx mRNA levels in all tested tissues were sharply elevated on the first day post-TMUV infection, and then lasted for at least three more days, especially in the brain, blood, spleen, and thymus (Figure 2). We do not know why goMx mRNA levels in the pancreas showed no significant difference at dpi3 and dpi4. However, all data here suggested that goMx is involved in the goose antiviral innate immune response.

### 3.2. Intracellular Location of the EGFP Mx Fusion Protein in Different Cell Lines

**Figure 3:**
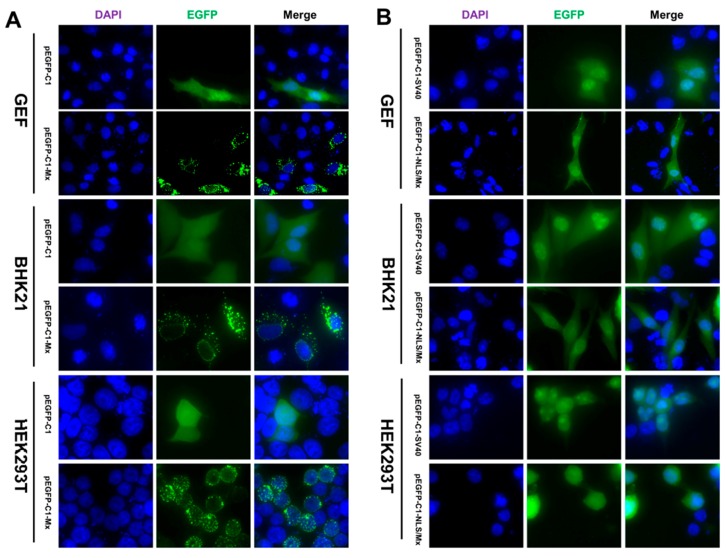
Cellular localization of the goMx protein. (**A**) Subcellular localization of pEGFP-C1-Mx in GEF, baby hamster kidney (BHK21), and human fetal kidney (HEK) 293T cells. Cells were transfected with indicated plasmids (pEGFP-C1-Mx or pEGFP-C1). At 24 h post-transfection (hpt), cells were fixed and stained with 4′,6-diamidine-2′-phenylindole dihydrochloride (DAPI). (**B**) The putative bipartite nuclear localization signal (NLS) is attributed to the nucleus localization of the fusion goMx protein. Localizations of pEGFP-C1-NLS/Mx in GEF, BHK21, and HEK 293T cells are shown, while pEGFP-C1-SV40 works as the positive control. The overexpressing protein appeared green, and the nucleus was stained blue with DAPI. Fluorescence was observed by fluorescence microscopy under a magnification of 400 times and analysed using Image Pro Plus 6.0.

To identify the exact location of goMx, we observed the distribution pattern of pEGFP-C1-Mx in GEFs, and in BHK21 and HEK 293T cells. We found that pEGFP-C1-Mx was distributed in a granular-like pattern in the cytoplasm and in a dot-like pattern in the nucleus of the transfected cells (GEFs, BHK21, and HEK 293T cells), which was strikingly different from the control pEGFP-C1, uniformly dispersed throughout the transfected cells (Figure 3A). The perinuclear regions seemed to contain the highest concentrations of the goMx fusion protein, and goMx accumulation in the nucleus was less than that in the cytoplasmic and perinuclear regions. Thus, the goMx protein in the nucleus (expressed from pcDNA3.1(+) plasmids) could barely be detected using the His-tagged antibody (Figure S1 in the Supplementary Material). As Figure S1 in the Supplementary Material shows, goMx variants of *Mx/K125A and K125A*Mx/ΔL4 aggregated in larger granules than the wild-type Mx, *Mx/T145A, and T145A*Mx/ΔL4, which formed more characteristic perinuclear assemblies. Resembling the wild-type goMx, the deletion variant of *Mx/ΔL4 showed a diffuse cytoplasmic staining pattern.

### 3.3. Nuclear Localization of goMx Depends on Its Predicted Bipartite NLS

As goMx is too large to passively diffuse, the only way it can access the nucleus is through active transport via the nuclear pore complex (NPC). Though lacking classical NLS bound with basic amino acids in its terminus, goMx possesses an NLS-like motif that is longer and rich in arginine and lysine (NENRLFAKLRKEFQTWGLILMENAAKVQKSI). Whether the putative NLS affects its nuclear-targeting ability or not aroused our interest. Considering the dispersed basic amino acids determining nuclear targeting, introducing site-directed mutagenesis into the goMx NLS is unavailable. To directly examine the relationship between the bipartite NLS and intracellular localization of the goMx, we fusion-expressed the putative bipartite NLS with EGFP (pEGFP-C1-NLS/Mx) in GEFs, and in BHK21 and HEK 293T cells, while pEGFP-C1-SV40 and pEGFP-C1 were used as positive and empty controls, respectively. Briefly, pEGFP-C1-SV40 and pEGFP-C1-NLS/Mx accumulated mainly within the nucleus, whereas pEGFP-C1 appeared green throughout the cell (Figure 3B).

### 3.4. Antiviral Activity of the goMx

**Figure 4:**
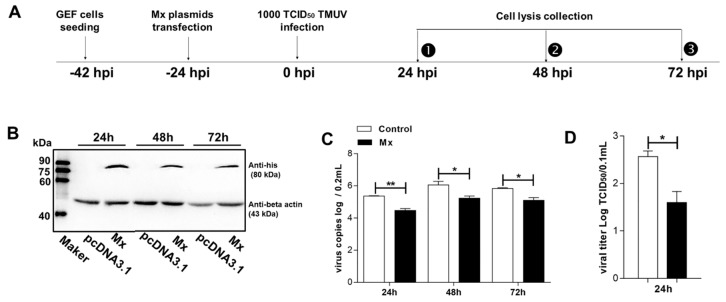
The antiviral activity of goMx. (**A**) Experimental procedure of the antiviral test. (**B**) Detection of goMx protein by western blot analysis. Cell extracts were prepared, and samples of 15 μL of protein per lane were separated by SDS-polyacrylamide gel electrophoresis. GoMx protein was detected with a His-tagged monoclonal antibody. (**C**) Inhibition of TMUV genomic RNA by goMx in GEF. At 24, 48, and 72 h post-infection (hpi), infected GEF were collected and lysed in 500 μL cell culture supernatants. Total RNA was extracted using 200 μL cell lysis and RT-qPCR was performed in triplicate. Data are shown as mean ± SD of three independent experiments (** *p* < 0.01, * *p* < 0.5). (**D**) TCID_50_ test. The infectious titers from culture supernatants were titrated using the TCID_50_ test from control GEF and goMx-expressing cells. The results showed the differences between both groups were significant at 24 hpi. Data are expressed as the mean ± SD of three independent experiments.

The high transcript levels of goMx against TMUV infection led us to examine whether or not exogenous Mx harbors antiviral activity against TMUV. According to the antiviral assay procedure (Figure 4A), the expression level of goMx was confirmed using western blotting, and the molecular mass was identified as 80 kDa (Figure 4B). In the antiviral assay, we collected GEFs at the indicated times, and demonstrated that TMUV was inhibited by goMx proteins from 24 hpi, at the early stage, to 72 hpi, at the late time point (Figure 4C). Although virus copies tended to slightly decrease owing to cell death at the late time point (72 hpi), consistently lower TMUV copies were detected in experimental cells rather than in the controls. Additionally, a TMUV titer assay was performed to quantify virus progeny. The virus titer derived from cells overexpressing goMx were significantly reduced by 1.2log10-fold at 24 hpi compared with those from control cells (Figure 4D). All our results confirmed the critical importance of goMx for TMUV resistance in vitro.

### 3.5. pEGFP-C1-Mx Fusion Protein Inhibited TMUV Replication

**Figure 5:**
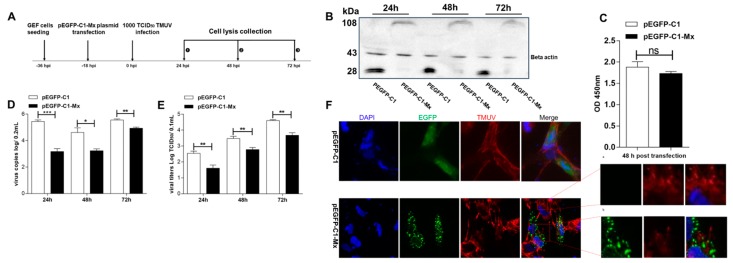
The pronounced inhibition of TMUV replication in GEF-expressing EGFP fusion goMx. (**A**) Experimental procedure of the antiviral test. (**B**) Detection of pEGFP fusion Mx protein by western blot analysis. Cell extracts were prepared, and samples of 15 μL of protein per lane were separated by SDS-polyacrylamide gel electrophoresis. (**C**) Viability of GEF cells transfected with pEGFP-C1 and pEGFP-C1-Mx. GEF cells were grown in a 96-well plate and transfected with pEGFP-C1 and pEGFP-C1-Mx. After 48 h, the cell viability was determined by CCK8 assay. Data are expressed as mean ± SD from three independent experiments. (**D**) Inhibition of TMUV genomic RNA by overexpressed pEGFP-C1-Mx. At 24, 48, and 72 hpi, infected GEFs were collected and lysed by three freezing–thawing cycles in a volume of 500 μL cell culture supernatants. RT-qPCR was performed in triplicate, and data are shown as mean ± SD of three independent experiments (*p*-value <0.05 and *p*-value <0.01 represent significant differences). (**E**) Inhibition of TMUV titers by overexpressed pEGFP-C1-Mx. The virus titer was determined by measuring the TCID_50_ directly on the cells expressing pEGFP-C1-Mx or pEGFP-C1 as outlined in experimental procedures. Data are expressed as the mean ± SD of three independent experiments. (**F**) Subcellular colocalization of goMx (in green) with TMUV (in red) in infected GEFs. GEF cells were transfected with pEGFP-C1-Mx and pEGFP-C1. At 18 hpt, cells were inoculated with TMUV for an additional 24 h. The coverslips were incubated with a mouse polyclonal antibody (1:500) recognizing TMUV in 1% bovine serum albumin (BSA) phosphate-buffered saline (PBS), washed in PBS, and then incubated with the Alexa Fluor PE-conjugated fluorescent goat-anti-mouse secondary antibodies (Life Technologies) for an additional 45 min. The fluorescence was detected by fluorescence microscopy with a 400× magnification. The enlarged picture indicates the cells with bare pEGFP-C1-Mx expression levels, but high TMUV loads (**a**), and the cells with high pEGFP-C1-Mx expression levels but very low TMUV loads (**b**). *** *p* < 0.001, ** *p* < 0.01, * *p* < 0.5.

To analyze the impact of EGFP on the antiviral activity of the goMx protein, we also tested the antiviral activity of the pEGFP-C1-Mx fusion protein. According to the antiviral assay procedure (Figure 5A), protein expression and cell viability were confirmed (Figure 5B,C). The pEGFP-C1-Mx overexpression could significantly inhibit TMUV replication in vitro. Consistent with the TMUV copies (Figure 5D), the titers of the progeny TMUV of pEGFP-C1-Mx-transfected cells significantly decreased from 24 hpi compared with control pEGFP-C1-expressing cells (Figure 5E). In addition, as shown in Figure 5F, cells with high viral loads (red fluorescent signals for TMUV) showed no green fluorescent signals (goMx low expression; Figure 5F(a)), while cells with high goMx expression (green fluorescent signals for goMx) barely contained any red fluorescent signals (low TMUV loads; Figure 5F(b)). More strikingly, pEGFP-C1-Mx proteins tended to translocate around the viral protein upon viral infection with TMUV. This finding intuitively confirmed the important activity of goMx against TMUV infection in the cytoplasm.

### 3.6. Antiviral Determinants of goMx

**Figure 6:**
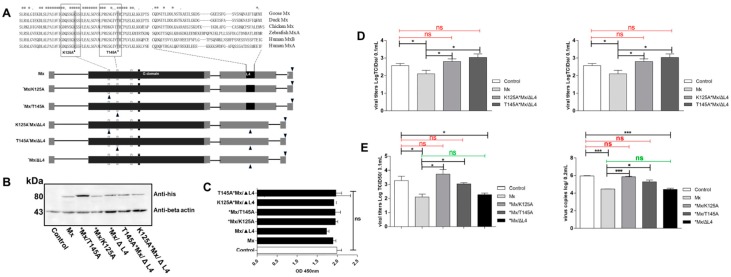
The antiviral determinants of goMx. (**A**) Construction of wild-type goMx and its variants, including the GTPase-deficient mutants, *Mx/K125A and *Mx/T145A, the *Mx/ΔL4 deletion plasmid, and two combination mutation plasmids of K125A*Mx/ΔL4 and T145A*Mx/ΔL4. The solid pillar stands for the Mg^2+^ active domain, while the blank pillar represents the tripartite GTP-binding domain consisting of GxxxxGKS, DxxG, and TKxD. The black triangles mark the point and deletion mutations, while the inverted black triangles represent the His-tag. (**B**) Detection of His-tagged wild-type goMx and its variants using western blot analysis. GoMx proteins and beta-actin were analyzed with western blot using an anti-His monoclonal antibody and anti-beta-actin antibody, respectively. The polyvinylidene fluoride (PVDF) membrane incubated with the goat-anti-mouse secondary antibodies. (**C**) Cell viability analysis of goMx and its variant protein in GEF. CCK8 assays of indicated plasmids were performed in triplicate. (**D**) The antiviral activity of goMx and its variants, K125A*Mx/ΔL4 and T145A*Mx/ΔL4, completely abolished antiviral activity. Transiently transfected GEF-expressing His-tagged Mx or its variant proteins, K125A*Mx/ΔL4 and T145A*Mx/ΔL4, were infected with 100 μL of 1000 TCID_50_ TMUV per cell, then the cells were collected by three-cycle freezing and thawing, using a volume of 500 μL PBS at different time points (24, 48, and 72 hpi). The inhibition ability of TMUV was determined by TCID_50_ and viral copy tests. Results are presented as means of technical duplicates of three independent experiments. (**E**) The antiviral activity of Mx and its variants. The K125 and T145 amino acids in the GTPase domain could account for deficient antiviral activity. Antiviral activity of the single substitution of *Mx/T145A and *K/125A or deletion *Mx/ΔL4 was assessed by TCID_50_ and viral copy tests. The statistical analysis was performed in GraphPad Prism using unpaired two-tailed *t*-tests: “ns” means no significance (the red “ns” is compared to the control, and the green “ns” is compared to the wild-type Mx as the experimental positive group; * *p* < 0.05; *** *p* < 0.001.).

To determine the key residues of goMx, we constructed a set of goMx variants (Figure 6A). Firstly, Western blot analysis confirmed that these proteins were successfully expressed (Figure 6B). GEFs were treated with the indicated plasmids for 36 h, and cell viability was assessed using a CCK8 assay (Figure 6C). As shown in Figure 6C, goMx and its variants showed no significant cytotoxicity. To examine the anti-TMUV activity of the goMx variants, we infected goMx variant-expressing GEFs with TMUV, and virus copies and TCID_50_ were further analyzed. Complete loss of antiviral activity (both virus copies and viral titers) of two mutants (K125A*Mx/ΔL4 and T145A*Mx/ΔL4) was observed (Figure 6D). To further identify which motif or specific amino acid (the L4 loop, K125, or T145) is related to the antiviral properties of goMx, the antiviral activities of *Mx/K125A, *Mx/T145A, and *Mx/ΔL4 against TMUV were investigated separately. Though the L4 loop of the goMx protein tolerates a great deal of sequence diversity (Figure 6A), L4 loop-truncated goMx (*Mx/ΔL4) did not lose its antiviral activity, representing no differences (*p* > 0.05) between the *Mx/ΔL4 and wild-type goMx-expressing cells (Figure 6E). However, we found that both variants, *Mx/K125A and *Mx/T145A, showed no antiviral activity against TMUV (in both copies and titers; Figure 6E). In total, both amino acid substitutions at position 125 (K) and 145 (T) of the GTP-binding motif, but not the L4 loop (40 residues: the 532nd–572nd amino acids), were vital for the antiviral activity of the goMx protein against TMUV in vitro.

## 4. Discussion

Birds are sensitive to some well-known flaviviruses, including TMUV, WNV, Japanese encephalitis (JEV), St. Louis encephalitis (SLEV), and Murray Valley encephalitis (MVEV) [37,38]. Although Mx is a well-known antiviral factor, the antiviral activity of bird Mx proteins against flaviviruses is unknown. Both in vitro and in vivo, it was shown that porcine Mx1 can confer resistance to classical swine fever virus belonging to the pestivirus genus in the flaviviridae family [39,40]. However, both porcine Mx1 and Mx2 proteins were recently found to be unable to inhibit Japanese encephalitis virus (JEV) proliferation [41]. Unlike the human Mx gene, which is highly conserved and possesses only a few single nucleotide polymorphisms [42,43], domestic animals possessing a highly polymorphic genome undoubtedly have differential antiviral activity [44,45,46]. Our previous study demonstrated that goose IFNs, combined with its ISGs, are involved in effectively controlling TMUV replication in vitro [35,36]. The antiviral effects of goMx and goose OASL (goOASL) were previously confirmed by transient overexpression and knockdown assays in vitro [35]. Here, we further investigated the subcellular localization patterns, antiviral characteristics, and antiviral determinants of goMx.

Rodent Mx1 proteins accumulate in the nucleus in characteristic dots closely associated with promyelocytic leukaemia (PML) nuclear bodies [47]. Mammalian Mx1 proteins are generally located in the cytoplasm in association with membranes of the smooth endoplasmic reticulum (ER) [48,49]. Unlike chicken Mx proteins, which are in a granular-like pattern and are exclusively extranuclear [50,51], goMx localized both in the cytoplasm and in the nucleus (Figure 3A), and resembled the small punctate bodies of human MxA and the distinct nuclear dot pattern of mouse Mx1. In the next set of fluorescent plasmids, the predicted NLS of the goMx protein showed clear nuclear-targeting capacity (Figure 3B). Additionally, since different interactors are required for the subcellular localization of Mx proteins [18,47,52], namely, the small ubiquitin-like modifier SUMO-1 and smooth ER marker syntaxin 17, we suggest that such partners must be conserved between mammalian and bird cells for the similar distribution pattern of goMx in GEFs, and in BHK21 and HEK 293T cell lines. Human MxA GTPase-deficient mutants, as exemplified for K83A and T103A (MxA^K83A^ or MxA^T103A^), showed GTP binding, but not hydrolysis, and aggregated in large cytosolic clusters. In agreement with this finding, we observed differences between wild-type goMx and its variants (Figure S1). A single alanine (Ala) substitution in the 125th (K) and 145th (T) amino acids can notably change the aggregation form, and the underlying molecular mechanism remains unknown. The nuclear loss of red fluorescent signals for wild-type goMx could be explained by both the insensitivity of the anti-His antibody and the lower expression of goMx.

It is worth mentioning that the intracellular location and antiviral activity of Mx proteins may be functionally associated. It was observed that the higher expression region of goMx in the cytoplasm showed lower TMUV viral loads (Figure 5F(a,b)). Excitingly, the colocalization of goMx with TMUV in the cytoplasm of infected GEFs stresses the importance of their distinct cytoplasm distribution to some degree. It was shown that a chimeric MxA protein with the NLS of rodent Mx1 can locate to the nucleolus and reduce human immunodeficiency virus (HIV) infection [53]. The C-terminal 91 amino acids of human MxB were also observed to promote accumulation at the nuclear envelope, thus conferring HIV-1 suppressor capabilities [54]. Considering that flavivirus replication is known to be exclusively cytoplasmic, we did not focus on the association between antiviral activity and nuclear goMx, although we identified a bipartite NLS important for the weak nuclear distribution of goMx.

The proposed mode of Mx action suggests that the dimeric or tetrameric forms of Mx might be released from the membrane-associated aggregates upon virus infection, and that GTP hydrolysis is, thus, a prerequisite for dissociating these assemblies [12,16,17]. The consequent conformational changes may lead to the immobilization, degradation, or redistribution of the viral targets, thereby inhibiting viral replication [20,55,56]. Human MxA^K83A^ and MxA^T103A^ mutants (single Ala amino acid substitution in the GTP-binding element), corresponding to goMx variants of K125A and T145A, formed some large intracellular aggregates [17]. Additionally, some reports suggested that human MxA^K83A^ or MxA^T103A^ and the equivalent mouse Mx1^K49A^ lost their antiviral function [17,57,58]. Based on these findings, we introduced Ala exchange on the same sites on GTP-binding elements of the goMx protein (*Mx/T145A or *Mx/K125A). While the L4 loop is at the equivalent sequence position as the pleckstrin homology (PH) domain of dynamin, and is predicted to be non-structured, it was previously shown to serve as a major antiviral-specificity determinant [12,17]. In our study, combination variants of K125A*Mx/ΔL4 and T145A*Mx/ΔL4 were both antiviral inactive (Figure 6D). To further characterize the relationship between the antiviral activity and those domains, a panel of mutants was tested for antiviral activity. We found that either the *Mx/T145A or *Mx/K125A mutant in two separate GTP-binding elements of the G domain lost its antiviral activity against TMUV in vitro (Figure 6E). The L4 loop is clearly not the motif that determines the antiviral activity of goMx against TMUV. Confirming our finding, a previous report demonstrated that the deletion of the L4 motif can still result in a stable dimer that is able to promote nuclear accumulation of the cytoplasmic wild-type MxA [12]. The L4 loop in human MxA and mouse Mx1 proteins serves as the major, but not the only, antiviral determinant [8,14]. In contrast, the N-terminus of human MxB, but not the L4 loop, is critical for HIV-1 restriction [17,54]. It is believed that the antiviral activity of the goMx protein against TMUV does not appear to be mediated by the L4 loop; however, the residues at the 125th (K) and 145th (T) positions in GTP-binding elements are important for mediating antiviral activity. Differences in the intracellular localization of goMx and its variants were observed (Figure S1), and further study could reveal the molecular mechanism underlying the protein distribution pattern and its association with antiviral specificity.

Collectively, our results suggest that goMx was mainly located in the cytoplasm, and was sporadically distributed in the nucleus in both primary (GEFs) and passaged cells (BHK21 and HEK 293T). The intracellular localization is attributed to the predicted bipartite NLS (30 residues: 441st–471st amino acids of the goMx). Intuitively, it seems that cells with higher goMx expression tend to have lower TMUV loads, as determined by an immunofluorescence assay, especially around the perinuclear and cytoplasm regions. Both amino acid substitutions at positions 125 (K) and 145 (T) of the GTP-binding element, not the L4 loop, were vital for the antiviral activity of goMx against TMUV in vitro.

## Figures and Tables

**Table 1:** Primer sequences for plasmid construction. The sequences in the parentheses represent the homologous arm sequences in the indicated vectors. In the pEGFP-C1-Mx-F primer (*), bases of lowercase letters are the hydrophobicity linker peptide (GGGGS)_3_. The nucleotides in red represent the six-His tag sequence.

Plasmid Name	Primer Name	Sequence
Mx	pCDNA3.1-Mx/his F	(CTAGCGTTTAAACTT)AAGCTTGCCACCATGTACCACAGAAGTCCC
	pCDNA3.1-Mx/his R	(TGCTGGATATCTGCA)GAATTCTCAGTGGTGGTGGTGGTGGTGCAGACAGCTAAAGGTCTT
pEGFP-C1-NLS/Mx	pEGFP-C1-NLS/Mx-F	(TCAGATCTCGAGCTC)AAGCTTCGAATGAAAACAGACTTTTTGC
	pEGFP-C1-NLS/Mx -R	(GGATCCCGGGCCCGC)GGTACCCTATATGCTTTTTTGAACCTT
pEGFP-C1-Mx	* pEGFP-C1-Mx-F	(TCAGATCTCGAGCTC) ggtggaggaggttctggaggcggtggaagtggtggcggaggtagcATGTACCACAGAAGTCCC
	pEGFP-C1-Mx-R	(GGATCCCGGGCCCGC) GGTACCAGGTGTTTGTGTGACTATGG
*Mx/K125A	*Mx/K125A-F1	AGCTCTGGGGCAAGCTCCA
	pCDNA3.1-Mx/his R	(TGCTGGATATCTGCA)GAATTCTCAGTGGTGGTGGTGGTGGTGCAGACAGCTAAAGGTCTT
	pCDNA3.1-Mx/his F	(CTAGCGTTTAAACTT)AAGCTTGCCACCATGTACCACAGAAGTCCC
	*Mx/K125A-R1	TGGAGCTTGCCCCAGAGCT
*Mx/T145A	*Mx/T145A-F1	GTATCGTTGCACGATGTCC
	pCDNA3.1-Mx/his R	(TGCTGGATATCTGCA)GAATTCTCAGTGGTGGTGGTGGTGGTGCAGACAGCTAAAGGTCTT
	pCDNA3.1-Mx/his F	(CTAGCGTTTAAACTT)AAGCTTGCCACCATGTACCACAGAAGTCCC
	*Mx/T145A-R1	GGACATCGTGCAACGATAC
*Mx/ΔL4	*Mx/ΔL4-F	TCTCACACGAAGGCCTATT
	pCDNA3.1-Mx/his R	(TGCTGGATATCTGCA)GAATTCTCAGTGGTGGTGGTGGTGGTGCAGACAGCTAAAGGTCTT
	pCDNA3.1-Mx/his F	(CTAGCGTTTAAACTT)AAGCTTGCCACCATGTACCACAGAAGTCCC
	*Mx/ΔL4-R	AATAGGCCTTCGTGTGAGAGTATACGATTCTCTCCATT
T145A*Mx/ΔL4	T145A*Mx/ΔL4-F	TCTCACACGAAGGCCTATT
	pCDNA3.1-Mx/his R	(TGCTGGATATCTGCA)GAATTCTCAGTGGTGGTGGTGGTGGTGCAGACAGCTAAAGGTCTT
	pCDNA3.1-Mx/his F	(CTAGCGTTTAAACTT)AAGCTTGCCACCATGTACCACAGAAGTCCC
	T145A*Mx/ΔL4-R	AATAGGCCTTCGTGTGAGAGTATACGATTCTCTCCATT
	T145A*Mx/ΔL4-F1	GTATCGTTGCACGATGTCC
	T145A*Mx/ΔL4-R1	GGACATCGTGCAACGATAC
K125A*Mx/ΔL4	K125A*Mx/ΔL4-F	TCTCACACGAAGGCCTATT
	pCDNA3.1-Mx/his R	(TGCTGGATATCTGCA)GAATTCTCAGTGGTGGTGGTGGTGGTGCAGACAGCTAAAGGTCTT
	pCDNA3.1-Mx/his F	(CTAGCGTTTAAACTT)AAGCTTGCCACCATGTACCACAGAAGTCCC
	K125A*Mx/ΔL4-R	AATAGGCCTTCGTGTGAGAGTATACGATTCTCTCCATT
	K125A*Mx/ΔL4-F1	AGCTCTGGGGCAAGCTCCT
	K125A*Mx/ΔL4-R1	AGGAGCTTGCCCCAGAGCT

## Supplementary Materials

The following are available online at http://www.mdpi.com/1999-4915/10/7/361/s1, Figure S1: Intracellular distribution of goMx variants. BHK21 cells were transfected with expression plasmids for wild goMx and its variants. At 24 h post-transfection, cells were fixed and stained with a His-tagged monoclonal antibody at a 1:500 dilution, and then incubated with the Alexa Fluor PE-conjugated fluorescent goat-anti-mouse secondary antibodies against wild goMx and its variants.
