# Supplementary material for: The 125th Lys and 145th Thr Amino Acids in the GTPase Domain of Goose Mx Confer Its Antiviral Activity against the Tembusu Virus
Source: Viruses. 2018 Jul 6;10(7):361. doi: 10.3390/v10070361 (PMC6070871; doi:10.3390/v10070361)
Supplement: Supplementary file 1 [file viruses-10-00361-s001.pdf]

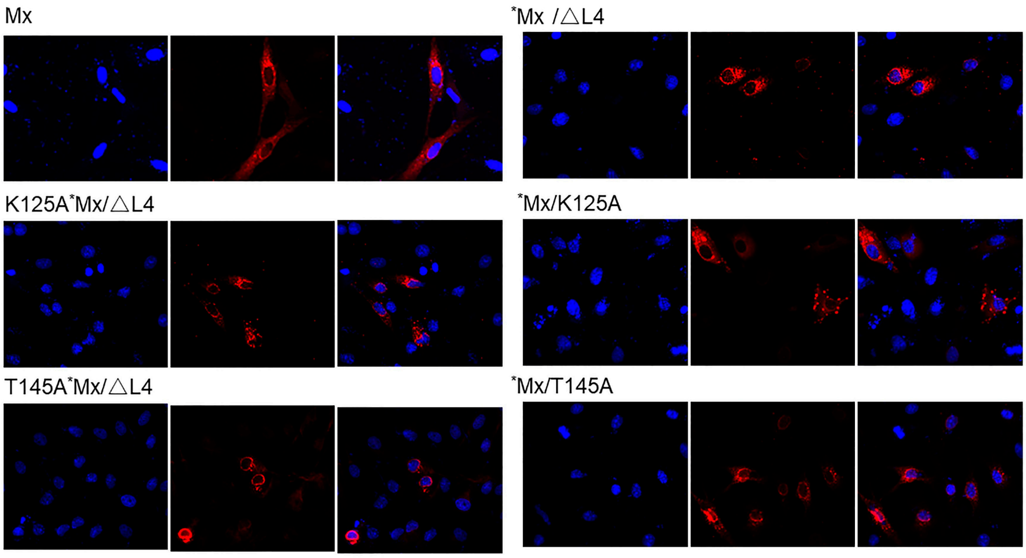

Figure S1. Intracellular distribution of goMx variants. BHK21 cells were transfected with expression plasmids for wild goMx and its variants. At 24 h post-transfection, cells were fixed and stained with a His-tagged monoclonal antibody at a 1:500 dilution, and then incubated with the Alexa Fluor PE-conjugated fluorescent goat-anti-mouse secondary antibodies against wild goMx and its variants.
